# Clinical and Epidemiological Characteristics of Rhabdomyolysis: A Retrospective Study

**DOI:** 10.1155/2023/6396576

**Published:** 2023-09-29

**Authors:** Keke Sun, Zhenhua Shi, Yushanjiang Abudureheman, Qihui Liu, Yibo Zhao, Xiangqun Zhang, Qi Lv, Yan Zhang, Songtao Shou, Heng Jin

**Affiliations:** ^1^Department of Emergency Medicine, Tianjin Medical University General Hospital, Tianjin, China; ^2^Department of Intensive Care Unit, PLA 983rd Hospital, Tianjin, China; ^3^Department of Critical Care Medicine, Hotan District People's Hospital, Xinjiang, China; ^4^Institute of Disaster and Emergency Medicine, Tianjin University, Tianjin, China

## Abstract

**Background:**

Rhabdomyolysis (RM) refers to a clinical syndrome in which muscle cells are damaged by various causes and the clinical manifestations are mainly muscle pain, weakness, and dark urine. Acute kidney injury (AKI) is a serious complication of RM with complex mechanisms and high mortality. Therefore, understanding the pathogenesis and clinical manifestations, early diagnosis and treatment of RM are crucial to improve its prognosis.

**Method:**

Analysis of medical records of RM patients admitted to Tianjin Medical University General Hospital from October 2019 to October 2022. Statistical software SPSS 25.0 was used to analyze the data. The risk factors of RM-complicated AKI were analyzed by logistic regression. The receiver operating characteristic (ROC) curve was plotted, the area under the curve (AUC) was calculated, and the optimal cutoff value was determined by the Youden index. *P* < 0.05 indicates a statistically significant difference between the groups.

**Result:**

Among the 71 patients, the median age of the patients was 53.0 (30.0, 71.0) years and was 2.5 times higher in men than in women. Infection was the most common etiology. History of alcohol consumption, CK, and creatinine were independent influencing factors for AKI due to RM. Logistic regression analysis showed that CK combined with creatinine had a better predictive value than the single index.

**Conclusion:**

Our study revealed the clinical and laboratory characteristics of RM in the population attending the Tianjin Medical University General Hospital in the last three years, which is a reference for future multicenter, prospective studies.

## 1. Introduction

Rhabdomyolysis (RM) is a clinical syndrome in which skeletal muscle is destroyed due to physiological, traumatic, genetic, toxic, or other causes, and muscle cytotoxic substances such as myoglobin and creatine kinase are released into the circulatory system, causing metabolic disorders and organ dysfunction [1]. Clinical manifestations of RM vary widely, from mildly elevated creatine kinase levels to life-threatening conditions associated with extremely elevated creatine kinase levels, electrolyte balance disturbances, and acute kidney injury (AKI) [2]. AKI is a serious complication of RM that can lead to serious health problems and even life-threatening conditions. Although RM has been widely studied, there are still some unknown factors, including the following aspects: some patients develop AKI, while the reason why other patients do not develop AKI is not clear; in some cases, myoglobin does not necessarily deposit in renal tubules leading to AKI even though creatine kinase levels are elevated, and the mechanism is not fully understood; further research is needed on the optimal treatment strategies and prevention methods for RM complicated with AKI. Through a retrospective study, to understand whether patients have AKI after the occurrence of rhabdomyolysis and its progress, we can determine the risk factors for RM complicated with AKI. Understanding these risk factors is helpful for screening high risk groups, prevention, and intervention in advance, so as to reduce the occurrence of AKI. Therefore, through a retrospective analysis, this study discussed the etiological distribution of RM, described the incidence and characteristics of RM complicated with AKI, and identified the factors related to the occurrence of AKI in order to provide evidence and guidance for clinical practice to improve the prognosis and quality of life of patients.

## 2. Methods

### 2.1. Study Design

This retrospective study analyzed the medical records of RM patients, who were diagnosed and treated at the Tianjin Medical University General Hospital from October 2019 to October 2022. This study was approved by the Medical Ethics Committee of Tianjin Medical University General Hospital. Informed consent was not required due to the retrospective nature of the study.

### 2.2. Study Population

**Figure 1:**
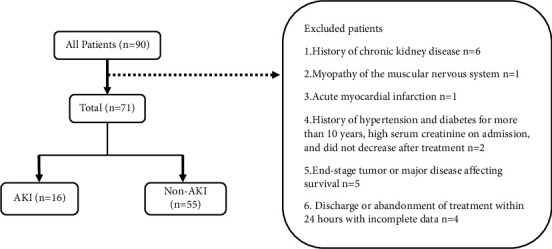
Flow diagram.

The inclusion criteria for this study were as follows: (1) the presence of causes and symptoms of muscle damage, such as muscle pain, weakness, and dark urine; (2) an increase in the serum creatine kinase (CK) level over five times the normal level; and (3) significant increase in the levels of myoglobin (MYO) in blood and urine. A total of 90 patients were eligible for inclusion. The exclusion criteria were as follows: (1) a history of chronic kidney disease; (2) a history of muscle and nervous system myopathy; (3) acute myocardial infarction; (4) a history of hypertension or diabetes mellitus for over 10 years with elevated serum creatinine on admission, which did not decrease significantly even after treatment; (5) patients with end-stage tumors or major diseases affecting survival; and (6) discharge or abandonment of treatment within 24 hours with incomplete data. All subjects (*n* = 71) were divided into AKI or non-AKI groups for comparison (Figure 1).

### 2.3. Data Collection

The medical records of patients were organized to record the gender, age, height, weight, symptoms, underlying disease, etiology and complications of rhabdomyolysis, vital signs, biochemical parameters (total protein, albumin, globulin, alanine aminotransferase, aspartate aminotransferase, lactate dehydrogenase, creatine kinase, creatine kinase isoenzyme, serum creatinine, uric acid, PH, potassium, sodium, calcium, lactic acid, myoglobin, urinary occult blood, white blood cells, red blood cells, hemoglobin, and platelets), treatment methods, and outcomes. According to the AKI clinical practice guidelines of the Kidney Disease Improvement Global Outcomes (KDIGO) organization [3], AKI is diagnosed if serum creatinine (SCr) ≥ 0.3 mg/dL (≥26.5 *µ*mol/L) within 48 h or SCr ≥ 1.5 times the basal value and is known or presumed to occur within 7 days, or the continuous urine output in 6 h is <0.5 mL/(kg·h). All laboratory tests were conducted on the day before admission or before performing any treatment after admission.

### 2.4. Statistical Analysis

The continuous variables between groups were compared using the Student *t*-test or Mann–Whitney *U* test, while categorical variables were compared using the chi-square test or Fisher's exact test. The statistical software SPSS 25.0 was used for the analysis. The normally distributed measurement data were represented as $\bar{x}\pm s$, while the non-normally distributed measurement data were represented as M (P25, P75). Logistic regression was used to analyze the risk factors for AKI complicated by rhabdomyolysis, to plot the subject operating characteristic (ROC) curve and calculate the area under the curve (AUC), and to determine the optimal cutoff value by the Youden index to maximize the sensitivity and specificity of the model. A *P* value <0.05 indicated statistically significant differences between the groups.

## 3. Results

### 3.1. Characteristics and Underlying Diseases

71 patients were included in the study according to the inclusion and exclusion criteria.

The median age of these patients was 53.0 (30.0, 71.0) years. There were 2.5 times more men than women (51 : 20). 2 (2.8%) patients were recurrent cases of rhabdomyolysis. 3 (4.2%) patients died. The median length of hospital days was 18.5 (9.0, 28.0) days in the AKI group and 10.0 (8.0, 15.0) days in the non-AKI group, with a statistically significant difference (*P* < 0.05). In addition, 50.0% of the patients with AKI had drinking history, 50.0% had oliguria or no uria symptoms, and 62.5% were accompanied by gastrointestinal symptoms of varying degrees (such as nausea, vomiting, and loss of appetite), which were statistically different from those without AKI (Table 1). In all patients, fatigue (56.3%) and tawny urine (54.9%) were the most common clinical manifestations, and myalgia (46.5%) was only found in less than half of the patients. In the analysis of underlying diseases, 50.0% of patients in the AKI group had previous neurological disease and only 27.3% of patients in the non-AKI group had a history of neurological disease, but this did not show a statistically significant difference, which may be related to the small number of cases in this study.  Table 2 lists the underlying diseases of each system in the patients in this study.

### 3.2. Etiology

**Figure 2:**
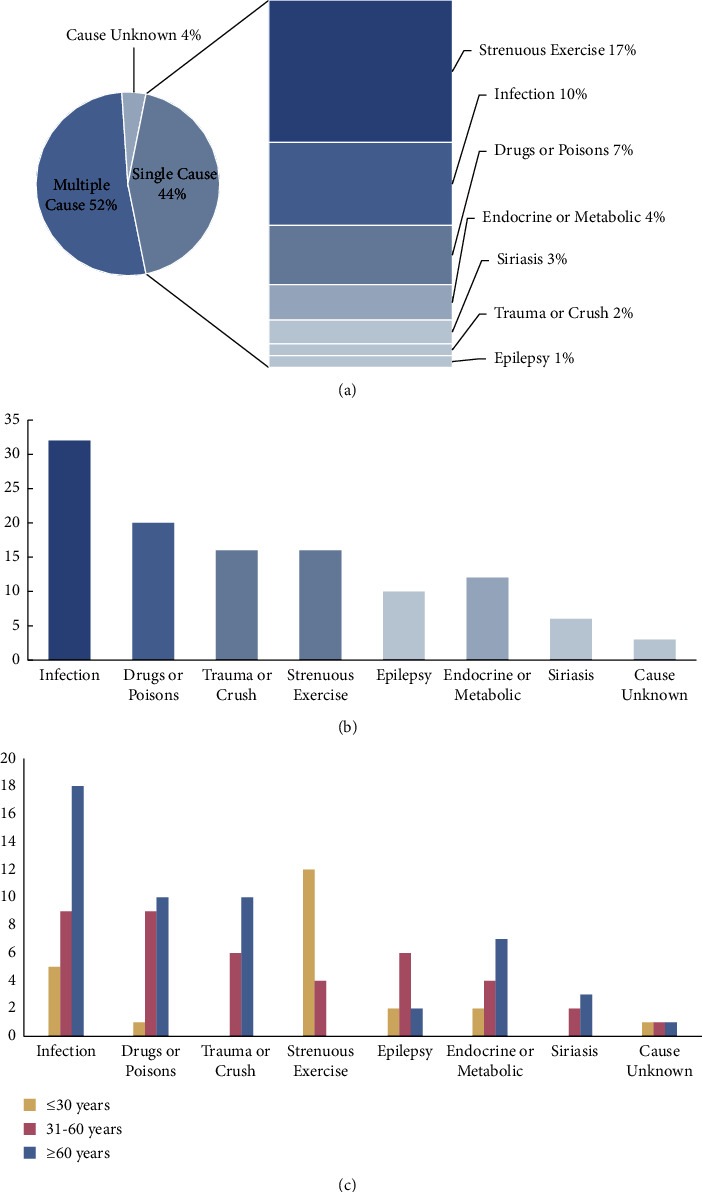
Etiology of rhabdomyolysis: (a) the proportion of single cause and multiple cause; (b) the number of people in each cause; (c) age distribution of etiology.

The etiology of RM is complex and diverse and can be divided into physical factors and nonphysical factors. The former mainly includes trauma, extrusion, strenuous exercise, seizure, and heat stroke, while the latter includes drugs, toxins, infect ions, endocrine diseases, and metabolic diseases.  Table 3 lists the detailed etiology in this study. Multiple etiologies that could lead to RM were present in most patients simultaneously. In this study, 31 (43.7%) patients had a single etiology, 37 (52.1%) patients had 2 or more possible etiological factors, and 3 (4.2%) patients had an unknown etiology. Infection was the most common cause of RM, with 32 (45.1%) patients causing RM due to infection or concurrent infection (Figure 2). Drugs and toxins were the next common causative agents, including alcohol intoxication, overconsumption of crayfish, and drug use. In the single cause analysis, strenuous exercise was the most common cause, with 83.3% of young adult males and 50% having the typical triad of “muscle pain, weakness, and dark urine.” In addition, some causes of RM are age-specific, with the highest frequency of strenuous exercise occurring in patients under 30 years of age and the highest frequency of infection occurring in those over 60 years of age, and RM due to drugs or poisons, trauma or crush, and endocrine or metabolic disorders increasing with age (Figure 2).

### 3.3. Laboratory Data

In the results of the univariate analysis (Table 4), ALT, AST, LDH, CK, SCr, URIC, MYO, WBC, and HGB were higher in the AKI group than in the non-AKI group, while calcium levels were lower in the RM-AKI group, and the differences were statistically significant (*P* < 0.05).

**Figure 3:**
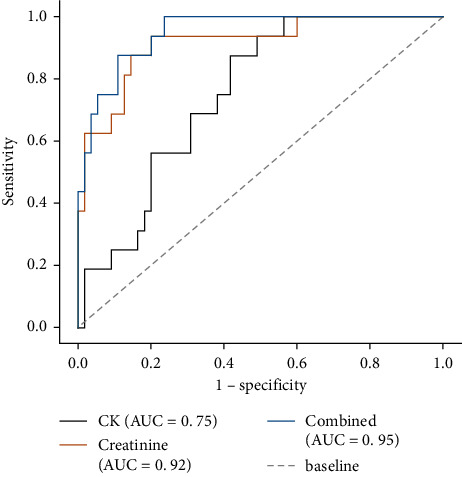
Receiver operating characteristic (ROC) curve analysis.

In the comparison between the two groups, ALT, CK, SCr, Ca, and WBC were significantly different (*P* < 0.01). As mentioned above, drinking history (*P* < 0.01) also had significant differences. Therefore, a binary logistic regression analysis was performed using whether AKI occurred as the dependent variable (AKI = 1, non-AKI = 0) and the above significantly different variables as independent variables. The results suggested that history of alcohol consumption, CK, and SCr were independent influencing factors for AKI in RM (Table 5). Logistic analysis was performed with CK and creatinine as independent variables to obtain predictive probability values, and the analysis was performed with the obtained predictive probability values as independent variables, and the results showed that the predictive value of CK combined with creatinine was better than that of a single indicator (Table 6 and Figure 3).

### 3.4. Complication

Only 18.3% of the 71 patients had no complications. 16 (22.5%) had AKI, 48 (67.6%) had varying degrees of hepatic impairment, and 32 (45.1%) had electrolyte metabolism disorders. The proportion of electrolyte disorders was higher in the AKI group, which was statistically different from the non-AKI group (*P* < 0.05) (Table 7). 3 (4.2%) patients died, all in the AKI group, and the causes of death were acute cerebral infarction, acute pulmonary embolism, and acute alcohol intoxication, respectively.

### 3.5. Treatment and Outcome

In this study, 56 (78.9%) patients received aggressive rehydration therapy, 41 (57.8%) patients received alkalinization of urine, and 10 (14.1%) patients received continuous renal replacement therapy (CRRT). Of the patients treated with CRRT, 4 died or abandoned treatment, and the remaining 6 had improved renal function after a mean of 9 ± 4.1 days of hemodialysis, and all renal function indexes returned to normal at discharge.

## 4. Discussion

In this study, among the total population with RM, males were 2.5 times more likely than females, which may be related to higher alcohol intake and smoking status in males, especially in RM due to strenuous exercise, where adolescent males accounted for 83.3%, revealing a male gender advantage. A survey of 235 patients with COVID-19 showed that [4] males and morbidly obese patients were independently associated with the development of RM, with males being an independent correlate of RM complicating AKI. Another study of 258 cases of RM due to salicylic acidosis showed that [5] men and seizures were risk factors for the development of RM. In addition, several studies have confirmed that the composition of RM etiology has age differences. In this study, the most common etiology in people under 30 years of age was exercise, and the most common etiology in people over 60 years of age was infection. Therefore, RM knowledge education should be conducted to prevent and identify RM early for different population characteristics.

In the analysis of underlying diseases, 32.4% of patients had previous neurological disorders, such as epilepsy, Parkinson's disease, and old cerebral infarction. On the one hand, epilepsy itself can be the cause of RM. Certain antiepileptic drugs (carbamazepine and levetiracetam) and lipid-lowering drugs (atorvastatin and rosuvastatin) taken orally in patients with epilepsy and old cerebral infarction have shown to be associated with the development of RM [6]. In a study of rhabdomyolysis in children, it was found that among patients with rhabdomyolysis complicated with AKI, the majority had an underlying condition, especially a neuropsychiatric condition, and was taking medication to treat it [7]. This is consistent with the results of this paper. Therefore, for those who have previous neurological diseases and long-term oral intake of drugs that can cause RM, creatine kinase and myoglobin should be detected regularly to be alert for the development of RM.

Infection was the primary cause of RM in our study. 45.1% of patients had infection, and in the AKI group, 63.5% of patients had infection of varying degrees. Bacterial infections were predominant, with the most common foci of infection being the lung, followed by the intestinal, intracranial, and urinary tracts. Pathogenic bacteria can directly invade myofibers and produce bacterial toxins that can directly cause damage to myocytes. However, the specific pathogens of infection were not statistically analyzed in this study. According to the literature, influenza virus, legionella, *Streptococcus pneumoniae*, and salmonella are common pathogens that cause RM [8, 9]. Therefore, infected individuals, especially those caused by influenza virus, legionella, and *Streptococcus pneumoniae*, should be alerted to the development of RM.

Among the 20 cases of RM due to drugs and poisons, 17 cases had a history of statins' use (mainly rosuvastatin and atorvastatin), 2 cases of alcoholism, and 1 case of excessive consumption of freshwater crayfish. RM caused by lipid-lowering drugs occurred mostly in patients with renal insufficiency, hypothyroidism, and infections and was induced by regular doses [10]. This may be related to its ability to cause disturbances in mitochondrial metabolism, increase intracellular calcium concentration, and affect cholesterol synthesis. Among the RM induced by statins in this study, there were 8 cases (47.1%) with concomitant infection or hypothyroidism. The incidence of statin-induced myopathy has been reported to be 3–100 per 20,000 patients [11]. Therefore, to avoid the occurrence of statin-related RM, clinicians should adhere to rational drug use, pay attention to avoid risk factors that induce RM, screen patients to assess thyroid function before starting statins, and closely observe patients' clinical manifestations and monitor laboratory test indexes such as CK during use to ensure the safety and effectiveness of the drugs. Alcohol can induce RM through different mechanisms, such as direct myotoxicity, fixation associated with compression and ischemic injury, or electrolyte abnormalities. In addition, the diuretic effect of alcohol can lead to dehydration and increase the risk of AKI. Chronic alcoholism also predisposes RM due to malnutrition, limited energy stores, and electrolyte abnormalities, which could explain the history of alcohol consumption embodied in our study as an independent risk factor for the development of AKI.

Strenuous exercise was also a major etiology. 15 (21.1%) patients were admitted with varying degrees of myalgia, malaise, and deepening of urine color after strenuous exercise. This group of patients contained mainly young adults, and the types of exercise were mainly long-distance running, swimming, and frog jumping. This is almost consistent with previous exercise-related rhabdomyolysis studies, in which patients are typically young exercisers who engage in high-intensity exercise, especially in hot or humid conditions, without adequate rest and hydration [12, 13]. 1 case was taking oral statin and beta-lipid-lowering drugs at the same time, 2 cases had hypokalemia at the same time, and the remaining 12 cases were caused by single etiology. 3 cases were complicated by AKI, but all renal function returned to normal after aggressive rehydration, alkalinization of urine, and symptomatic treatment. All patients in this group were discharged after an average of 6.5 ± 2.7 days of hospitalization. This is related to the fact that the patients are mostly young and strong, without underlying diseases and with strong renal compensatory capacity. In summary, attention should be paid to gradual exercise, avoiding sudden high-intensity training, and once symptoms such as muscle pain, weakness, and deepening of urine color appear after strenuous exercise, prompt medical consultation should be made, and early active rehydration should be done to prevent the occurrence of AKI.

In addition to the abovementioned causes, endocrine or metabolic diseases are also causes of RM. Among the 12 cases of RM due to endocrine or metabolic diseases, there were 6 cases of hypokalemia, 3 cases of acute complications of diabetes mellitus with diabetic ketoacidosis, 2 cases of primary hypothyroidism, and 1 case of hyperthyroidism. The presentation of these patients was usually atypical, with only 2 patients presenting with the classical triad. Therefore, these patients are more likely to be missed and misdiagnosed and need to be highly alerted by clinicians.

**Figure 4:**
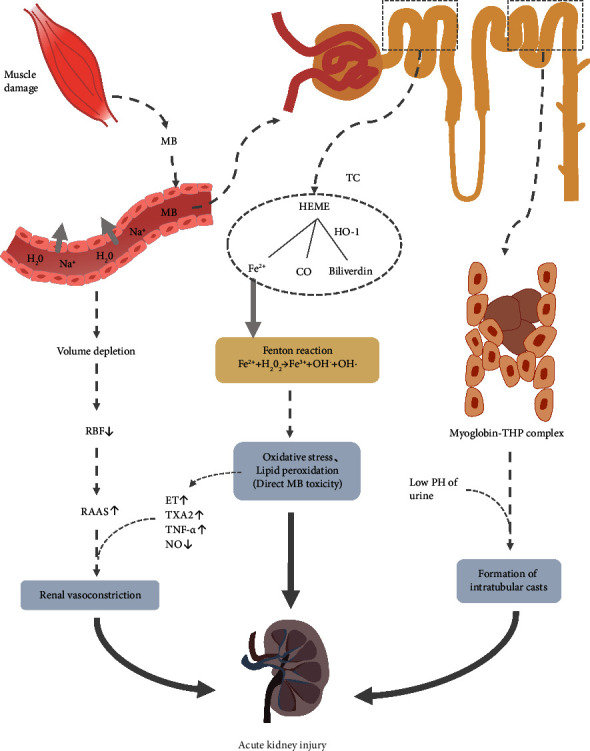
Pathophysiology of rhabdomyolysis-included acute kidney injury. MB: myoglobin; CO: carbon monoxide; HO-1: heme oxygenase-1; Fe^2+^: ferrous iron; Fe^3+^: ferric iron; H_2_O_2_: hydrogen peroxide; OH^−^: hydroxyl anion; OH: hydroxyl radical; ET: endothelin; TXA2: thromboxane A2; TNF-*α*: tumor necrosis factor *α*; NO: nitric oxide; RAAS: renin-angiotensin-aldosterone system; RBF: renal blood flow; TC: tubular cell; THP: Tamm–Horsfall protein.

AKI is a serious complication of RM. It has been reported in the literature that the incidence of AKI in RM is 13%–50% [14]. 16 (22.5%) patients in this study had concomitant AKI, which may be related to differences in AKI definition, patient cohort, or inclusion criteria. AKI secondary to RM has a high mortality rate and complex mechanisms. Myoglobin, the main pathogenic substance in RM concomitant with AKI, can be caused by renal vasoconstriction, proximal tubule injury, distal tubule obstruction, and other mechanisms to play a nephrotoxic effect [15, 16]. In the early stage of the disease, body fluids are retained in the interstitial spaces of the injured muscle tissue, reducing the blood volume and decreasing the renal blood flow, which in turn leads to the activation of the renin-angiotensin-aldosterone system (RAAS), resulting in constriction of the renal vasculature, one of the main mechanisms causing renal injury. Simultaneously, myoglobin-induced oxidative damage can increase the release of vasoconstrictors such as endothelin (ET), thromboxane A2 (TXA2), and tumor necrosis factor *α* (TNF-*α*) [17], as well as decrease the release of vasodilators such as NO [18], thereby aggravating renal vasoconstriction, reducing glomerular filtration rate, and causing AKI. In renal tubular epithelial cells, ferromyoglobin can be oxidized to ferric ions while generating free radicals, which can promote the lipid peroxidation of cell membranes and increase the generation of ROS [19], which in turn can lead to the apoptosis of renal tubular epithelial cells, resulting in renal damage. Myoglobin is a small molecule pigmented protein that is freely filtered by the glomerulus and enters the renal tubular epithelial cells by endocytosis, where it is metabolized [20]. Under normal physiological conditions, the deposition and toxic effects of myoglobin on the body will not occur. When RM occurs, the release of a large amount of myoglobin exceeds the binding capacity of plasma haptoglobin. When the free myoglobin flows through the distal tubule, it will combine with the Tamm–Horsfall protein to form a complex and deposit locally, thereby blocking the renal tubules and leading to AKI [21] (Figure 4). In summary, when RM occurs, any factor that reduces renal blood flow may aggravate the condition, and myoglobinuria is often more likely to lead to acute tubular necrosis and the development of substantial renal AKI. AKI complicated by RM is not uncommon in clinical practice, and it is easy to miss or misdiagnose the disease because of its diverse etiology, inconspicuous clinical manifestations, and varying degrees of severity.

For the comparison of whether AKI occurred, our study revealed significant differences in most conventional biomarkers. A review by Safari et al. reported a significant correlation between serum CK levels and RM-induced AKI [22], and our study is consistent with it; that is, higher serum CK level is associated with AKI. However, the screening performance characteristics of CK values in predicting the occurrence of AKI are not ideal (AUC = 0.75), and the exact threshold of CK remains a controversial issue. Therefore, it seems that triage and screening of patients based on CK levels alone are not very accurate. In our study, serum CK combined with creatinine evaluation was found to be helpful for accurate determination of patients with RM combined with AKI, with higher sensitivity and specificity, and better predictive value than a single index.

As mentioned previously, MYO plays a dominant role in the pathogenesis of RM-induced AKI, and it has been shown that peak myoglobin is a better predictor of AKI than serum CK [23]. However, this was not reflected in our study, which may be related to the data we collected, where when MYO levels exceeded the upper reference limit, only the values that did not exceed the limit were reported, not the exact values, which may lead to an underestimation of its impact. In this study, it was also found that ALT and AST levels were more significantly elevated in the AKI group, which may suggest that patients are more likely to have concomitant liver injury. However, it has also been suggested that in the case of RM, the source of the transaminase elevation is usually unclear [24]. Therefore, transaminase abnormalities may not indicate liver injury and need to be identified by clinicians in conjunction with medical history and other laboratory tests. It has been reported in the literature that LDH levels are higher in patients with renal impairment, and LDH is significantly higher in patients with RM [25]. In our study, LDH was higher in the AKI group than in the non-AKI group, but more studies are needed to confirm whether it can be used for the early diagnosis of RM-AKI. Our study also showed that WBC counts were higher in the AKI group than in the non-AKI group due to the presence of varying degrees of infection in 10 patients (62.5%) in the AKI group, which may lead to a more severe inflammatory response than in the non-AKI group. Although not directly related to the pathophysiology of RM, the WBC counts may reflect the severity and physiological stress associated with the underlying etiology; further studies are needed to determine the role of WBC counts in RM-induced AKI.

At present, there is a lack of randomized controlled studies on the treatment of RM in clinical practice, and certain treatment measures are still controversial. The accepted treatment principles include removal of the cause as soon as possible, massive fluid replacement as early as possible, and prevention and treatment of critical complications. Intravenous rehydration is an undisputed treatment for RM, and as discussed earlier, reduced blood volume is the main cause of RM causing AKI. Studies have found that patients who maintain polyuria within 6 hours after admission can successfully prevent AKI. At the early stage, 1.5 L/h of normal saline can be given to maintain the urine output at 200−300 mL/h. In this study, 56 (78.9%) patients received aggressive rehydration therapy after admission to the hospital. In addition, AKI caused by RM is associated with a decrease in urinary pH. Therefore, sodium bicarbonate can be used to alkalinize the urine to prevent nephrotoxic metabolites from myoglobin catabolism. However, it is important to note that large doses of bicarbonate may aggravate hypocalcemia and deposition of calcium salts in tissues. In this study, more than half of the patients (57.8%) were given alkalinizing urine treatment. Mannitol is an osmotic diuretic originally thought to promote increased renal blood flow, reduce tubular cast, and act as a free radical scavenger. However, there is no randomized controlled trial evidence that sodium bicarbonate or mannitol is more effective than aggressive fluid resuscitation alone in reducing acute kidney injury, the need for dialysis, or death. According to the available literature, mannitol, sodium bicarbonate, and furamide should not be routinely used for rhabdomyolysis. In this study, the population in the non-AKI group showed significant relief of muscle soreness in 2-3 d after treatment with removal of etiology, active rehydration, and maintenance of electrolyte balance. According to the degree of muscle injury in different patients, although the recovery time of CK, CK-MB, MYO, AST, SCr, and other laboratory test indicators are different, the prognosis is good. Although the above treatment is preferred, continuous renal replacement therapy (CRRT) should be considered for acute renal failure with severe hyperkalemia, metabolic acidosis, and oliguria [26]. In this study, 10 patients received CRRT. After comprehensive treatment, 6 patients improved and were discharged with normal renal function, and 4 patients failed to prevent further deterioration and were discharged automatically or died after receiving CRRT, with a mortality rate of 40%. Although receiving CRRT resulted in improvements in SCr and electrolyte levels, there is still room for improvement in the efficacy of CRRT in the treatment of critically ill RM patients.

Since this study was a retrospective single-center study, there were some limitations. First, only patients in the past three years were included in the study, and the sample size was relatively small. Second, some data on the etiology, history, and laboratory indicators were missing, leading to the exclusion of some cases. Third, AKI and oliguria may be underestimated or overestimated due to issues in the methodology, involving the measurement of baseline SCr levels and urine output. Fourth, the analytical methods and equipment used by different sections of the laboratory may vary, which may cause errors in the statistical results. Our study revealed the clinical and laboratory characteristics of RM among patients at the Tianjin Medical University General Hospital in the past three years, explored the characteristics and distribution of RM caused by different etiologies, described the incidence and characteristics of AKI concomitant with RM, and identified the factors associated with its occurrence. These findings will contribute to a wider understanding of RM, facilitate the early diagnosis and treatment of RM, prevent the occurrence of serious complications, and provide a reference for future multicenter and prospective studies.

## Figures and Tables

**Table 1:** Characteristics of patients with RM with or without AKI.

Category	Total (*n* = 71)	AKI (*n* = 16)	Non-AKI (*n* = 55)	*P* value
Gender				0.058
Male	51 (71.8)	15 (93.8)	36 (65.5)	
Female	20 (28.2)	1 (6.2)	19 (34.5)	
Age (year)	53.0 (30.0, 71.0)	61.0 (37.3, 67.8)	47.0 (29.0, 74.0)	0.052
Length of stay (day)	10.0 (8.0, 17.0)	18.5 (9.0, 28.0)	10.0 (8.0, 15.0)	0.030
Height (m)	1.72 (1.64, 1.76)	1.75 (1.68, 1.78)	1.71 (1.63, 1.75)	0.123
Weight (kg)	70.8 (57.0, 80.0)	80.0 (62.5, 89.3)	68.8 (52.0, 80.0)	0.059
Smoking history	19 (26.8)	7 (43.8)	12 (21.8)	0.155
Drinking history	14 (19.7)	8 (50.0)	6 (10.9)	0.002
Clinical manifestation				
Muscle pain	33 (46.5)	7 (43.8)	26 (47.2)	0.804
Weakness	40 (56.3)	10 (62.5)	30 (54.5)	0.572
Dark urine	39 (54.9)	12 (75.0)	27 (49.1)	0.067
Oliguria or anuria	11 (15.5)	8 (50.0)	3 (5.5)	<0.001
With GI symptoms	28 (39.4)	10 (62.5)	18 (32.7)	0.032
Underlying disease				
Hypertension	33 (46.5)	9 (56.3)	24 (43.6)	0.373
Diabetes mellitus	11 (15.5)	3 (18.8)	8 (14.5)	0.987
Digestive system	9 (12.7)	0 (0)	9 (16.4)	0.192
Respiratory system	8 (11.3)	0 (0)	8 (14.5)	0.242
Circulatory system	18 (25.4)	3 (18.8)	15 (27.3)	0.716
Nervous system	23 (32.4)	8 (50.0)	15 (27.3)	0.087
Mental system	6 (8.5)	1 (6.3)	5 (9.1)	>0.999
Endocrine and metabolic	10 (14.1)	1 (6.3)	9 (16.4)	0.538
Vital signs				
T (°C)	36.6 (36.5, 36.9)	36.7 (36.5, 37.2)	36.5 (36.4, 36.8)	0.185
P (times/min)	80.0 (74.0, 89.0)	84.0 (72.3, 91.8)	80.0 (74.0, 89.0)	0.762
R (times/min)	18.0 (18.0, 20.0)	20.5 (18.0, 25.8)	18.0 (17.0, 20.0)	0.016
Systolic BP (mmHg)	141.0 (118.0, 157.0)	152.5 (137.3, 164.8)	135.0 (117.0, 156.0)	0.113
Diastolic BP (mmHg)	75.0 (66.0, 86.0)	76.0 (66.3, 89.5)	75.0 (65.0, 83.0)	0.675

Data are expressed as number (%) or median (P25, P75). RM: rhabdomyolysis; AKI: acute kidney injury; GI: gastrointestinal; T: temperature; P: pulse; R: respiration; BP: blood pressure.

**Table 2:** The underlying diseases of the patients.

System	Diseases
Digestive system	Duodenal ulcer, cholecystolithiasis, fatty liver, hepatitis, reflux esophagitis, and helicobacter pylori infection
Respiratory system	Bronchial asthma, chronic obstructive pulmonary disease, atelectasis, and sleep apnea syndrome
Circulatory system	Obsolete myocardial infarction, coronary heart disease, arrhythmia, pulmonary artery embolism, and pulmonary heart disease
Nervous system	Obsolete cerebral infarction, cerebral atrophy, cerebral hemorrhage, Parkinson's disease, epilepsy, and febrile convulsion
Mental system	Anxiety disorder, depressive disorder, sleep disorders, and hypophrenia
Endocrine and metabolic	Hyperlipidemia, gout, hyperthyroidism, hypothyroidism, and hypopituitarism

**Table 3:** List of causes in the study.

Category	Cause
Infection	Intestinal infection, pneumonia, urinary tract infection, intracranial infection, cholecystitis, and erysipelas
Drugs or poisons	Alcoholism, cray, and medicine (statins, fibrates, and antiepileptic)
Trauma or crush	Tumble and traffic accident
Strenuous exercise	Long-distance running, swimming, and leapfrog
Epilepsy	Grand mal and convulsion
Endocrine or metabolic	Hypokalemia, diabetic ketosis, hypertonic hyperglycemic syndrome, and hypothyroidism
Siriasis	

**Table 4:** Laboratory results of patients with RM with or without AKI.

Category	Total (*n* = 71)	AKI (*n* = 16)	Non-AKI (*n* = 55)	*P* value
Urine occult blood				0.061
—	19 (26.8)	2 (12.5)	17 (30.9)	
1+	6 (8.5)	0 (0)	6 (10.9)	
2+	4 (5.6)	2 (12.5)	2 (3.6)	
3+	41 (57.7)	12 (75.0)	29 (52.7)	
TP (g/L)	68.0 (63.0, 75.0)	67.0 (63.0, 71.8)	68.0 (62.0, 76.0)	0.563
Albumin (g/L)	38.0 (35.0, 41.0)	38.0 (34.0, 39.5)	39.0 (35.0, 43.0)	0.087
Globulin (g/L)	30.0 (27.0, 34.0)	30.5 (24.3, 33.8)	30.0 (27.0, 34.0)	0.945
ALT (U/L)	80.0 (44.0, 215.0)	241.5 (119.8, 281.8)	60.0 (40.0, 148.0)	0.002
AST (U/L)	208.0 (97.0, 595.0)	476.5 (153.0, 1277.8)	168.0 (92.0, 534.0)	0.026
LDH (U/L)	635.0 (348.0, 1595.0)	1248.0 (523.8, 3968.0)	585.0 (317.0, 1423.0)	0.025
CK (U/L)	15399.0 (4655.0, 34563.0)	32723.5 (16081.5, 56319.5)	11158.0 (4235.0, 27475.0)	0.002
CK-MB (U/L)	272.0 (98.0, 1026.0)	669.5 (185.5, 1394.8)	216.0 (77.0, 993.0)	0.058
Creatinine (*μ*mol/L)	79.0 (54.0, 210.0)	390.5 (208.3, 580.3)	69.0 (49.9, 109.0)	<0.001
Uric acid (*μ*mol/L)	383.0 (231.0, 558.0)	635.5 (375.8, 937.5)	358.0 (226.0, 512.0)	0.017
pH	7.40 (7.36, 7.43)	7.39 (7.32, 7.42)	7.40 (7.38, 7.43)	0.191
Potassium (mmol/L)	3.70 (3.10, 4.20)	4.02 (3.53, 4.56)	3.67 (3.00, 4.10)	0.055
Sodion (mmol/L)	138.4 (133.0, 142.1)	135.2 (128.1, 140.2)	139.0 (135.0, 142.9)	0.096
Calcium (mmol/L)	2.10 (2.00, 2.24)	1.97 (1.68, 2.18)	2.15 (2.00, 2.30)	0.006
Lac (mmol/L)	1.5 (1.0, 2.3)	1.2 (1.0, 1.9)	1.7 (1.0, 2.5)	0.352
Troponin T (ng/ml)	0.044 (0.007, 0.195)	0.050 (0.011, 0.813)	0.040 (0.006, 0.147)	0.223
MYO (ng/ml)	1707.0 (418.0, 3000.0)	3000.0 (1729.1, 3000.0)	1237.0 (395.1, 2815.2)	0.011
WBC (*∗*10 ^ 9/L)	9.55 (6.88, 16.81)	13.63 (9.68, 16.25)	8.57 (6.23, 12.07)	0.001
RBC (*∗*10 ^ 9/L)	4.44 (4.00, 4.90)	4.48 (4.16, 5.15)	4.40 (3.98, 4.88)	0.474
HGB (g/L)	135.0 (120.0, 152.0)	144.0 (137.3, 157.5)	133.0 (119.0, 144.0)	0.023
PLT (*∗*10 ^ 9/L)	211.0 (174.0, 272.0)	200.0 (155.5, 229.0)	217.0 (181.0, 281.0)	0.152

Data are expressed as number (%) or median (P25, P75). RM: rhabdomyolysis; AKI: acute kidney injury; TP: total protein; ALT: alanine aminotransferase; AST: aspartate aminotransferase; LDH: lactate dehydrogenase; CK: creatine kinase; pH: pondus hydregenii; MYO: myohemoglobin; WBC: white blood cell; RBC: red blood cell; HGB: hemoglobin; PLT: platelet.

**Table 5:** The logistic regression analysis of RM complicated with AKI.

Category	*β*	SE	Wald *χ*^2^	*P* value	OR	OR 95% CI
Drinking history	−5.962	2.957	4.065	0.044	0.003	(0.000∼0.847)
ALT	0.001	0.004	0.046	0.831	1.001	(0.993∼1.009)
CK	0.000	0.000	4.848	0.028	1.000	(1.000∼1.000)
Creatinine	0.021	0.010	4.367	0.037	1.021	(1.001∼1.041)
Calcium	−10.056	6.279	2.565	0.109	0.000	(0.000∼9.490)
WBC	0.474	0.245	3.732	0.053	1.606	(0.993∼2.591)

RM: rhabdomyolysis; AKI: acute kidney injury; ALT: alanine aminotransferase; CK: creatine kinase; WBC: white blood cell.

**Table 6:** The predictive value of CK, creatinine, and combined testing for RM-AKI.

Category	AUC (95% CI)	Cutoff	Sensitivity (%)	Specificity (%)	Youden (%)
CK	0.751 (0.635∼0.867)	12945.5	87.5	58.2	0.457
Creatinine	0.915 (0.834∼0.996)	112.50	93.8	80.0	0.738
Combined testing	0.949 (0.901∼0.997)	—	87.5	89.1	0.766

RM: rhabdomyolysis; AKI: acute kidney injury; CK: creatine kinase; AUC: area under the curve.

**Table 7:** Complications of RM in patients with or without AKI.

Complications	Total (*n* = 71)	AKI (*n* = 16)	Non-AKI (*n* = 55)	*P* value
None	13 (18.3)	0 (0)	13 (23.64)	0.074
Liver function damage	48 (67.6)	13 (81.25)	35 (63.64)	0.185
Electrolyte disturbance	32 (45.1)	11 (68.75)	21 (38.18)	0.031
Death	3 (4.2)	3 (18.75)	0 (0)	0.010

RM: rhabdomyolysis; AKI: acute kidney injury.

## Data Availability

The data used to support the findings of this study are available on request.
